# Insomnia in Breast Cancer: A Neglected Symptom Cluster

**DOI:** 10.3390/jcm15124603

**Published:** 2026-06-13

**Authors:** Giuseppe Marano, Ida Paris, Gianandrea Traversi, Osvaldo Mazza, Antonella Migliore, Valentina Ricozzi, Silvia Rotondaro, Francesco Pavese, Tatiana D’Angelo, Paola Fuso, Alessandra Fabi, Gianluca Franceschini, Marianna Mazza

**Affiliations:** 1 Department of Neuroscience, Head-Neck and Chest, Section of Psychiatry, Fondazione Policlinico Universitario Agostino Gemelli IRCCS, Largo Agostino Gemelli 8, 00168 Rome, Italy; 2 Department of Neuroscience, Section of Psychiatry, Università Cattolica del Sacro Cuore, 00168 Rome, Italy; 3 Division of Gynecologic Oncology, Department of Woman and Child Health and Public Health, Fondazione Policlinico Universitario Agostino Gemelli IRCCS, 00168 Rome, Italyvalentina.ricozzi@guest.policlinicogemelli.it (V.R.);; 4 Unit of Medical Genetics, Department of Laboratory Medicine, Ospedale Isola Tiberina-Gemelli Isola, 00186 Rome, Italy; gianandrea.traversi@gmail.com; 5 Spine Surgery Department, Bambino Gesù Children’s Hospital IRCCS, 00168 Rome, Italy; osvaldo.mazza1973@hotmail.it; 6 Precision Medicine Unit in Senology, Fondazione Policlinico Universitario Agostino Gemelli IRCCS, Largo Agostino Gemelli 8, 00168 Rome, Italy; alessandra.fabi@policlinicogemelli.it; 7 Breast Surgery Unit, Department of Woman and Child’s Health and Public Health Sciences, Fondazione Policlinico Universitario Agostino Gemelli IRCCS, 00168 Rome, Italy; gianluca.franceschini@policlinicogemelli.it; 8 Department of Medical and Surgical Sciences, Catholic University of the Sacred Heart, 00168 Rome, Italy

**Keywords:** breast cancer, insomnia, sleep disturbances, psycho-oncology, neuropsychiatry, inflammation, circadian rhythm, depression

## Abstract

**Background/Objectives**: Insomnia is one of the most prevalent and persistent symptoms among patients with breast cancer, yet it remains under-recognized and undertreated in routine clinical practice. Beyond its impact on sleep quality, insomnia is increasingly understood as a multidimensional condition involving neurobiological, psychological, and behavioral mechanisms, closely intertwined with cancer-related stress and psychiatric comorbidities. This narrative review aims to provide a comprehensive and integrative overview of insomnia in breast cancer, focusing on its epidemiology, pathophysiological underpinnings, neuropsychiatric correlates, and clinical implications, while highlighting gaps in current research and management. **Methods**: A narrative review of the literature was conducted, including studies published in major medical databases (PubMed, Scopus, and Web of Science) up to 2025. Relevant articles addressing insomnia, sleep disturbances, psychiatric symptoms, and neurobiological mechanisms in breast cancer populations were selected and synthesized. **Results**: Insomnia affects a substantial proportion of breast cancer patients across the disease trajectory, from diagnosis to survivorship. Its etiology is multifactorial, involving dysregulation of the hypothalamic–pituitary–adrenal axis, inflammatory processes, and circadian rhythm, as well as treatment-related factors such as chemotherapy, endocrine therapy, and menopausal symptoms. Insomnia frequently co-occurs with depression, anxiety, fatigue, and pain, forming symptom clusters that significantly impair quality of life and may influence clinical outcomes. Emerging evidence supports a bidirectional relationship between insomnia and psychiatric vulnerability, suggesting a shared neurobiological substrate within the brain–body stress axis. **Conclusions**: Insomnia in breast cancer should be conceptualized as a neuropsychiatric condition embedded within a broader stress-related symptom network rather than as an isolated sleep disturbance. Improved screening, interdisciplinary management, and the integration of evidence-based interventions such as cognitive behavioral therapy for insomnia are essential. Research should focus on personalized and mechanistically informed approaches to better address this highly prevalent yet insufficiently managed condition.

## 1. Introduction

Breast cancer is the most commonly diagnosed malignancy among women worldwide and, for a growing proportion of patients, is increasingly associated with prolonged survivorship due to advances in screening, early detection, systemic therapies, surgery, radiotherapy, and supportive care. However, the term “chronic condition” should be interpreted with caution, as the clinical trajectory of breast cancer varies substantially according to disease stage, biological subtype, treatment responsiveness, metastatic status, and survivorship context. In this review, we use this concept primarily to refer to the expanding population of women living for years after diagnosis and treatment, often with persistent physical, neuropsychological, and behavioral sequelae. Alongside improved survival, there has been increasing recognition of the long-term symptom burden associated with breast cancer and its treatments, including psychological distress, fatigue, cognitive complaints, menopausal symptoms, pain, and sleep disturbances [1,2].

Insomnia is defined as persistent difficulty in initiating or maintaining sleep, or experiencing non-restorative sleep, accompanied by daytime impairment. While its prevalence in the general population ranges between 6% and 10%, substantially higher rates have been consistently reported in oncological populations, especially among women with breast cancer [3]. Indeed, insomnia affects up to 42–69% of patients with breast cancer, with even higher rates reported during active treatment and survivorship phases [3]. For example, endocrine therapy and taxane chemotherapy have been defined as independent risk factors for sleep disorders in patients with breast cancer [4].

Poor sleep quality may affect more than 60% of breast cancer survivors, reflecting a clinically significant and persistent symptom across the disease trajectory [2,5,6].

Importantly, insomnia in breast cancer does not occur in isolation but rather within a complex network of co-occurring symptoms, including depression, anxiety, fatigue, pain, and cognitive impairment. This clustering suggests shared underlying mechanisms and has led to the conceptualization of insomnia as part of a broader symptom cluster with significant implications for quality of life and functional outcomes [7,8,9]. In particular, insomnia has been identified as both a consequence and a potential driver of depressive symptomatology, with evidence supporting a bidirectional relationship mediated, at least in part, by inflammatory and neuroendocrine pathways [10]. Furthermore, clinical studies have demonstrated strong associations between insomnia severity and cognitive dysfunction in breast cancer survivors, highlighting its impact on higher-order brain functions [11].

From a pathophysiological perspective, insomnia in breast cancer is increasingly understood as a multifactorial condition involving the interplay of biological, psychological, and treatment-related factors. Dysregulation of the hypothalamic–pituitary–adrenal (HPA) axis, alterations in inflammatory signaling, and disruption of circadian rhythms have all been implicated in the development and maintenance of sleep disturbances in this population [2,12]. In parallel, cancer-related treatments, including chemotherapy, endocrine therapy, and radiation, contribute to sleep disruption through mechanisms such as hormonal deprivation, vasomotor symptoms, pain, and fatigue [3]. In female patients, hormonal disruption plays a central role, as oncological treatments are often the cause of iatrogenic menopause, leading to vasomotor symptoms, mood and cognitive disturbances, sexual dysfunction, and genitourinary complaints, all contributing to sleep disruption [13,14]. 

Psychological stress related to cancer diagnosis, fear of recurrence, and existential distress further amplifies vulnerability to insomnia, reinforcing its characterization as a stress-related disorder embedded within the brain–body axis.

Despite its high prevalence and clinical relevance, insomnia remains insufficiently addressed in routine oncological care. Evidence-based interventions, particularly cognitive behavioral therapy for insomnia (CBT-I), have demonstrated robust efficacy in improving sleep outcomes and associated psychological symptoms in breast cancer populations [3]. These interventions are still underutilized, and screening for sleep disorders is not systematically implemented in many clinical settings.

In this context, a comprehensive and integrative understanding of insomnia in breast cancer is critically needed. This narrative review aims to examine insomnia through a neuropsychiatric lens, integrating current evidence on epidemiology, biological mechanisms, psychiatric comorbidities, and clinical implications. By framing insomnia as a central component of a broader stress-related symptom network, this review seeks to highlight the need for interdisciplinary approaches and to identify potential directions for personalized and mechanism-based interventions.

## 2. Materials and Methods

This article was designed as a narrative review aimed at synthesizing current evidence on insomnia in breast cancer from a neuropsychiatric perspective, with particular attention to epidemiology, pathophysiological mechanisms, psychiatric correlates, symptom clustering, and treatment implications. The review was conducted following principles of transparency, methodological clarity, and focused literature appraisal recommended for high-quality narrative reviews [15,16].

A literature search was performed in PubMed/MEDLINE, Scopus, and Web of Science to identify relevant studies published from January 2020 to March 2026. Additional seminal articles were considered when necessary to contextualize key concepts or support methodological statements. The search strategy combined controlled vocabulary and free-text terms related to breast cancer and insomnia, including: “breast cancer”, “breast neoplasms”, “insomnia”, “sleep disturbance”, “sleep disorders”, “psycho-oncology”, “depression”, “anxiety”, “fatigue”, “inflammation”, “circadian rhythm”, “cognitive behavioral therapy for insomnia”, and “survivorship”. Boolean operators (“AND”, “OR”) were used to refine the search.

Eligible publications included narrative reviews, systematic reviews, meta-analyses, observational studies, interventional studies, and relevant clinical papers addressing insomnia or clinically meaningful sleep disturbances in adult patients with breast cancer during active treatment or survivorship. Studies focused exclusively on other cancer populations, pediatric samples, or non-clinically relevant sleep outcomes were excluded unless they contributed to the conceptual framing of neuropsychiatric mechanisms.

Articles were screened based on relevance, recency, methodological robustness, and consistency with the aims of the review. Particular priority was given to the recent literature, especially studies published in the last five years, in order to provide an updated overview of the field, in line with current recommendations for narrative reviews [17]. Reference lists of selected articles were also hand-searched to identify additional pertinent publications.

**Figure 1:**
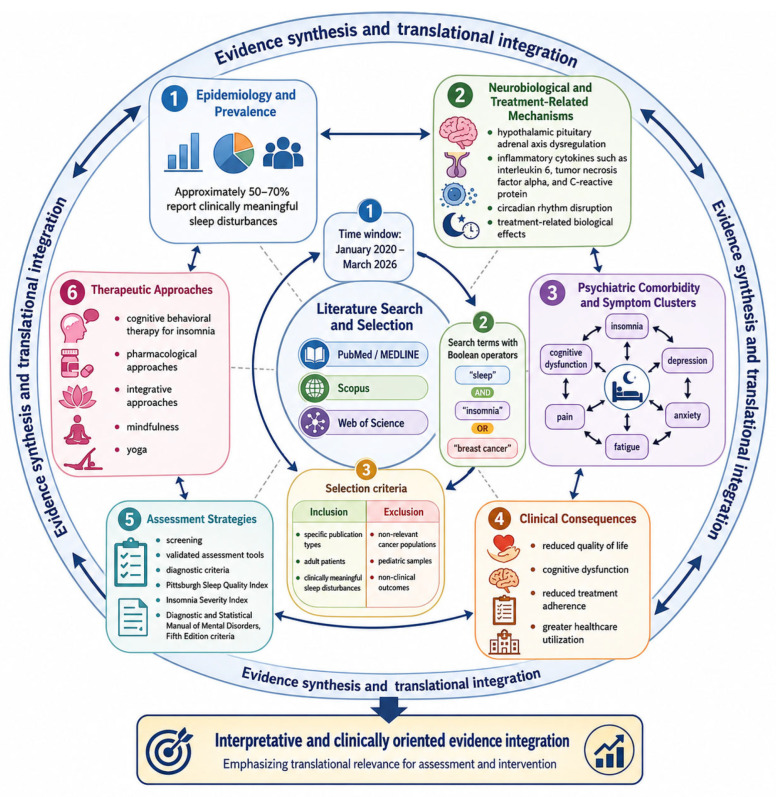
Conceptual map of the review methodology and thematic organization. Note: This circular diagram illustrates the structured workflow of the narrative review. The central core depicts the methodological process, starting with the Literature Search & Selection across three databases (PubMed/MEDLINE, Scopus, Web of Science). Flowing arrows indicate the timeline (January 2020–March 2026), key search terms integrated with Boolean operators (AND, OR), and the Selection Criteria nested within: green half for Inclusion (specific publications, adult patients, clinically meaningful disturbances), and red half for Exclusion (non-relevant cancers, pediatric samples, non-clinical outcomes). Radiating outwards from the core are distinct, interconnected thematic domains of the evidence synthesis: Epidemiology & Prevalence (50–70% clinically meaningful sleep disturbances reported); Neurobiological & Treatment-Related Mechanisms (hypothalamic-pituitary-adrenal axis dysregulation, cytokines interleukin-6, tumor necrosis factor-alpha, C-reactive protein, circadian rhythm disruption); Psychiatric Comorbidity & Symptom Clusters (insomnia, depression, anxiety, fatigue, pain, cognitive dysfunction bidirectional loops); Clinical Consequences (reduced quality of life, cognitive dysfunction, treatment adherence, healthcare utilization); Assessment Strategies (screening, tools, diagnostic criteria, e.g., Pittsburgh Sleep Quality Index, Insomnia Severity Index); Therapeutic Approaches (cognitive behavioral therapy for insomnia, pharmacological, integrative, e.g., mindfulness, yoga). The outermost ring and final outcomes box culminate in Interpretative & Clinically Oriented Evidence Integration, emphasizing translational relevance.

The included literature was narratively synthesized and organized into the following thematic domains: epidemiology and prevalence; neurobiological and treatment-related mechanisms; psychiatric comorbidity and symptom clusters; clinical consequences; assessment strategies; and therapeutic approaches. To facilitate a comprehensive understanding of the methodology underpinning this narrative review, including the process of literature identification, selection, and thematic organization, a detailed conceptual map has been developed and is presented in Figure 1, providing a structured visual representation of the review framework.

In keeping with recommendations for narrative reviews, the objective was not to produce a pooled quantitative estimate but rather to provide an interpretative and clinically oriented integration of the evidence, with emphasis on the translational relevance of insomnia as a neuropsychiatric condition in breast cancer.

## 3. Epidemiology and Clinical Burden

Sleep disturbances are among the most prevalent and burdensome symptoms experienced by patients with breast cancer across all phases of the disease trajectory, from diagnosis to long-term survivorship. Recent evidence indicates that between 50% and 70% of women with breast cancer report clinically significant sleep disturbances, with insomnia representing the most common and persistent subtype [18]. Notably, prevalence rates vary depending on the assessment tools and diagnostic criteria used, but consistently exceed those observed in the general population.

A large meta-analysis conducted on cancer populations reported that approximately 62% of patients experience poor sleep quality, while clinically relevant insomnia symptoms are present in up to one-third of cases [19]. In breast cancer specifically, longitudinal studies suggest that insomnia symptoms often emerge at the time of diagnosis and may persist for years after completion of treatment, highlighting the chronic nature of sleep disruption in this population [20]. Survivorship cohorts have shown that sleep disturbances remain highly prevalent even 5–10 years post-treatment, suggesting that insomnia should be conceptualized as a long-term sequela rather than a transient treatment-related side effect.

Insomnia rarely occurs in isolation. Instead, it is frequently embedded within a broader symptom cluster that includes fatigue, pain, depression, and anxiety. This clustering has been consistently observed in recent oncological and psycho-oncological studies, supporting the hypothesis of shared underlying mechanisms and reinforcing the clinical relevance of insomnia as a central node within a multidimensional symptom network [21]. Notably, insomnia has been identified as a potential driver of symptom amplification, contributing to the persistence and severity of fatigue and emotional distress.

From a clinical standpoint, the burden of insomnia in breast cancer extends beyond sleep impairment alone. Poor sleep has been associated with reduced quality of life, impaired cognitive functioning, decreased treatment adherence, and increased healthcare utilization [18]. Emerging evidence also suggests a potential link between sleep disruption and adverse oncological outcomes, including altered immune function and possibly reduced survival, although causal relationships remain to be fully elucidated [22,23].

Despite its high prevalence and significant impact, insomnia remains underdiagnosed and undertreated in routine oncological care. Recent studies indicate that sleep disturbances are often not systematically assessed during clinical visits and, when identified, are frequently managed with pharmacological approaches alone, without integration of evidence-based behavioral interventions [24,25]. This gap between prevalence and clinical management underscores the need for increased awareness and structured screening strategies.

It can be argued that insomnia represents a highly prevalent, persistent, and clinically consequential condition in breast cancer, warranting greater attention within both oncological and psychiatric frameworks.

Although this review focuses on breast cancer, insomnia is not exclusive to this population and has been reported across several oncological diagnoses, including lung, gastrointestinal, gynecological, hematological, and prostate cancers. However, its prevalence and persistence may vary according to cancer type, disease stage, symptom burden, treatment exposure, endocrine disruption, pain, fatigue, and psychological distress. Breast and gynecological cancer populations appear particularly vulnerable, partly because treatments may induce abrupt hormonal changes, vasomotor symptoms, and menopausal complaints, which directly interfere with sleep continuity. More advanced disease, active treatment, higher pain burden, fatigue, fear of recurrence, and comorbid anxiety or depression may further increase insomnia severity. Therefore, insomnia should be conceptualized as a transdiagnostic oncological symptom, while recognizing that breast cancer provides a particularly relevant clinical model because of the combined effects of survivorship, endocrine manipulation, treatment-related toxicity, and psycho-oncological vulnerability [18,19,20,21].

**Figure 2:**
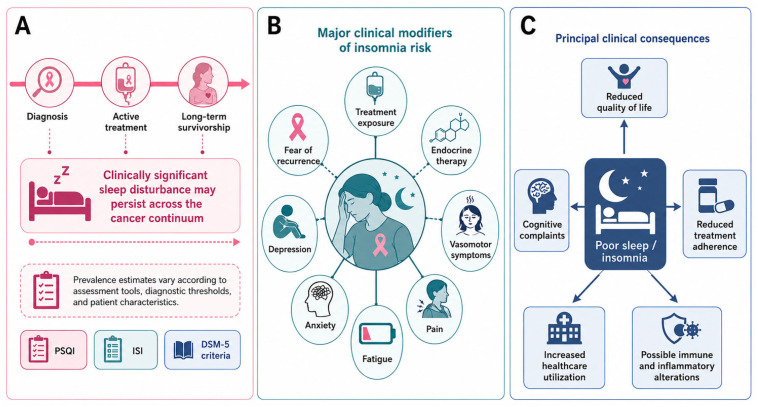
Epidemiological and clinical burden of insomnia in breast cancer. Note: figure summarizes three complementary aspects of sleep disturbance in breast cancer. (**A**) illustrates the persistence of clinically significant sleep disturbance from diagnosis and active treatment to long-term survivorship, while emphasizing that prevalence estimates vary according to assessment tools, diagnostic thresholds, and patient characteristics. (**B**) identifies major clinical modifiers of insomnia risk, including treatment exposure, endocrine therapy, vasomotor symptoms, pain, fatigue, anxiety, depression, and fear of recurrence. (**C**) summarizes the principal clinical consequences associated with poor sleep, including reduced quality of life, cognitive complaints, reduced treatment adherence, increased healthcare utilization, and possible immune and inflammatory alterations. Abbreviations: PSQI, Pittsburgh Sleep Quality Index; ISI, Insomnia Severity Index; DSM-5, Diagnostic and Statistical Manual of Mental Disorders, Fifth Edition.

Recognizing insomnia as a core component of the cancer-related symptom burden is a critical step toward improving patient-centered outcomes and developing targeted, multidisciplinary interventions. To provide a clearer depiction of insomnia and sleep disturbance across the breast cancer trajectory, Figure 2 summarizes three distinct but complementary dimensions: prevalence over time, major clinical modifiers, and downstream clinical consequences.

## 4. Pathophysiological and Neurobiological Mechanisms

Insomnia in breast cancer is increasingly conceptualized as a multifactorial condition arising from the interaction of neurobiological, psychological, and treatment-related mechanisms. Rather than representing a simple secondary symptom, sleep disturbance appears to reflect a broader dysregulation of the brain–body stress system, involving neuroendocrine, inflammatory, and circadian pathways. The co-occurrence of insomnia with depression, anxiety, fatigue, pain, and cognitive dysfunction is supported by convergent biological and psychological mechanisms rather than by simple symptom overlap. At the biological level, chronic stress and cancer-related inflammation may dysregulate the HPA axis, increase evening cortisol secretion, alter cytokine signaling, and disrupt circadian rhythmicity, all of which are relevant to both sleep regulation and mood vulnerability. At the psychological level, cancer-related threat appraisal, fear of recurrence, rumination, conditioned arousal, and maladaptive sleep behaviors may perpetuate insomnia while also increasing vulnerability to anxiety and depressive symptoms. Treatment-related factors, including chemotherapy, endocrine therapy, estrogen deprivation, vasomotor symptoms, pain, and fatigue, may further amplify this shared pathway. These mechanisms support the interpretation of insomnia as a clinically meaningful component of a broader neuropsychiatric symptom network rather than as a merely secondary complaint [26,27,28,29,30,31,32,33].

### 4.1. Neuroendocrine Dysregulation and HPA Axis Activation

One of the most consistently implicated mechanisms in cancer-related insomnia is the dysregulation of the HPA axis. Chronic psychological stress associated with cancer diagnosis and treatment may lead to sustained hyperactivation of the HPA axis, resulting in elevated cortisol levels and altered diurnal rhythms [26,27]. This disruption has been associated with impaired sleep initiation and maintenance, as well as with increased nocturnal arousal.

In breast cancer populations, flattened diurnal cortisol slopes and elevated evening cortisol levels have been linked to both sleep disturbances and poorer clinical outcomes, suggesting that neuroendocrine dysregulation may serve as a shared biological substrate for insomnia and disease progression [28]. More recent studies further support the role of stress-related neuroendocrine alterations in mediating the relationship between psychological distress and sleep disruption [26,27].

### 4.2. Inflammation and Immune Dysregulation

Inflammatory processes represent another central pathway linking breast cancer and insomnia. Cancer and its treatments are associated with increased production of pro-inflammatory cytokines, including interleukin-6 (IL-6), tumor necrosis factor-alpha (TNF-α), and C-reactive protein (CRP), which have been shown to directly influence sleep regulation [29,30]. In addition, a diet with pro-inflammatory potential has been correlated with sleep disorders among patients with breast cancer, which might be mediated by circulating interleukin-1β, interleukin-10, IL-6, TNF-α, and CRP [31].

Recent evidence indicates that elevated inflammatory markers are associated with both insomnia severity and fatigue in breast cancer patients, supporting the concept of a shared inflammatory basis for symptom clustering [32]. Moreover, inflammation-related alterations in central nervous system functioning, particularly within sleep-regulating brain regions, may contribute to hyperarousal and disrupted sleep architecture.

It is important to outline that the relationship between sleep and inflammation is bidirectional: while inflammation can impair sleep, chronic insomnia may further exacerbate inflammatory activation, creating a self-perpetuating cycle with potential implications for both psychological well-being and oncological outcomes [29,33].

### 4.3. Circadian Rhythm Disruption

Circadian dysregulation is a key mechanism underlying insomnia in breast cancer. Disruption of the central circadian clock, located in the suprachiasmatic nucleus, may result from both behavioral and biological factors, including irregular sleep–wake schedules, light exposure, and treatment-related changes in hormonal signaling [34].

Breast cancer treatments, such as chemotherapy and endocrine therapy, can significantly alter circadian rhythms, contributing to sleep fragmentation and reduced sleep efficiency. In addition, circadian disruption has been linked to tumor biology, including alterations in cell cycle regulation, DNA repair, and immune function, suggesting a potential role in cancer progression [35].

Research highlights the clinical relevance of circadian misalignment as both a contributor to insomnia and a potential therapeutic target. Interventions targeting circadian regulation, including light-based approaches, structured sleep–wake scheduling, physical activity, and chronotherapy-informed behavioral strategies, have shown preliminary or moderate benefits in oncology settings, particularly on sleep quality, sleep efficiency, fatigue, and circadian rest-activity patterns; however, findings remain heterogeneous, and additional breast cancer-specific trials using standardized sleep outcomes are needed before firm conclusions can be drawn [34,35].

### 4.4. Treatment-Related and Hormonal Factors

Breast cancer treatments play a substantial role in the development and maintenance of insomnia. Chemotherapy is frequently associated with fatigue, pain, and neurotoxicity, all of which may interfere with sleep regulation. Endocrine therapies, such as tamoxifen and aromatase inhibitors, induce estrogen deprivation, leading to vasomotor symptoms, particularly hot flashes and night sweats, that are strongly associated with nocturnal awakenings [36].

Treatment-related insomnia is not merely a transient side effect but may persist long after treatment completion, suggesting long-term neurobiological and hormonal alterations [37,38]. These findings further support the need to conceptualize insomnia as a chronic condition in breast cancer survivorship.

### 4.5. Psychological Stress and Central Nervous System Hyperarousal

Psychological factors represent a critical component in the pathophysiology of insomnia in breast cancer. The experience of cancer diagnosis is often associated with acute and chronic stress, fear of recurrence, and existential concerns, all of which contribute to heightened cognitive and emotional arousal [39].

From a neurobiological perspective, this state of hyperarousal is associated with increased activation of cortical and limbic structures, including the amygdala and prefrontal cortex, which play key roles in emotional regulation and sleep–wake control. Functional neuroimaging studies suggest that insomnia is characterized by persistent central nervous system activation, even during sleep, further supporting its conceptualization as a disorder of hyperarousal [40,41].

In breast cancer patients, the interaction between psychological stress and biological vulnerability appears particularly relevant, as stress-related neural circuits may amplify both sleep disturbance and psychiatric comorbidity [42].

**Figure 3:**
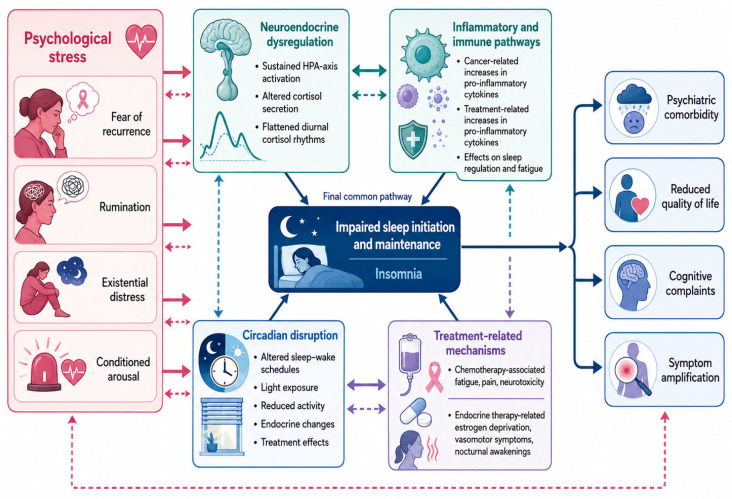
Integrated biological and psychological mechanisms underlying insomnia in breast cancer. Note: The figure presents insomnia as the final common pathway of interacting biological, psychological, and treatment-related processes. Psychological stress, including fear of recurrence, rumination, existential distress, and conditioned arousal, is represented as an upstream and bidirectionally interacting factor that may activate or amplify neuroendocrine dysregulation, inflammatory signaling, circadian disruption, and central nervous system hyperarousal. Neuroendocrine dysregulation includes sustained HPAaxis activation, altered cortisol secretion, and flattened diurnal cortisol rhythms. Inflammatory and immune pathways include cancer-related and treatment-related increases in pro-inflammatory cytokines, which may affect both sleep regulation and fatigue. Circadian disruption may result from altered sleep–wake schedules, light exposure, reduced activity, endocrine changes, and treatment effects. Treatment-related mechanisms include chemotherapy-associated fatigue, pain, neurotoxicity, and endocrine therapy-related estrogen deprivation; vasomotor symptoms; and nocturnal awakenings. These pathways converge on impaired sleep initiation and maintenance and may contribute to psychiatric comorbidity, reduced quality of life, cognitive complaints, and symptom amplification. Abbreviations: HPA axis, hypothalamic–pituitary–adrenal axis.

Insomnia in breast cancer emerges as the result of a complex interplay between neuroendocrine, inflammatory, circadian, treatment-related, and psychological factors. This integrative framework supports the conceptualization of insomnia as a neuropsychiatric condition embedded within the broader brain–body stress axis, with important implications for both clinical management and future research. Figure 3 presents an integrated mechanistic model in which psychological stress is represented as an upstream and bidirectionally interacting contributor to neuroendocrine, inflammatory, circadian, and treatment-related pathways. This structure avoids treating psychological stress as a completely separate pathway and instead emphasizes its role in amplifying biological vulnerability, central hyperarousal, and symptom persistence.

## 5. Psychiatric Comorbidity and Symptom Clusters

**Figure 4:**
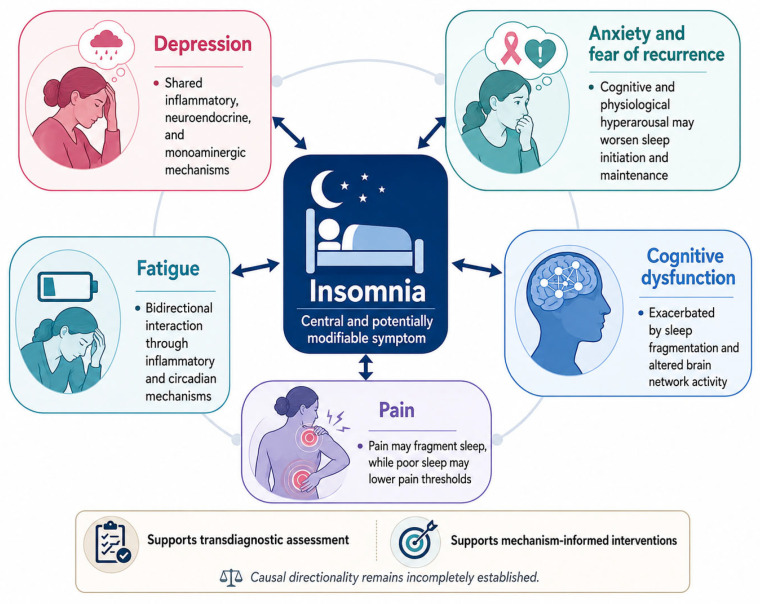
Neuropsychiatric symptom network centered on insomnia in breast cancer. Note: The figure presents insomnia as a potentially central and modifiable symptom within a broader network that includes depression, anxiety, fatigue, pain, and cognitive dysfunction. Single bidirectional arrows indicate reciprocal relationships and symptom amplification, rather than separate opposing pathways. Depression and insomnia may share inflammatory, neuroendocrine, and monoaminergic mechanisms. Anxiety and fear of recurrence may promote cognitive and physiological hyperarousal, thereby worsening sleep initiation and maintenance. Fatigue and insomnia may interact through inflammatory and circadian mechanisms. Pain may fragment sleep, while poor sleep may lower pain thresholds. Cognitive dysfunction may be exacerbated by sleep fragmentation and altered brain network activity. This network-based model supports transdiagnostic assessment and mechanism-informed interventions, while acknowledging that causal directionality remains incompletely established.

Insomnia in breast cancer is rarely an isolated clinical entity and is more appropriately conceptualized as part of a broader network of interrelated symptoms, including depression, anxiety, fatigue, pain, and cognitive dysfunction. This constellation of co-occurring symptoms, commonly referred to as a “symptom cluster,” reflects shared biological and psychological mechanisms and has significant implications for patient outcomes [43,44]. To illustrate the centrality of insomnia within a broader symptom network, a detailed representation of interrelated psychiatric and somatic symptoms is presented in Figure 4, highlighting potential targets for transdiagnostic interventions.

### 5.1. Insomnia and Depression

Depression is one of the most frequent psychiatric comorbidities in patients with breast cancer, with prevalence estimates ranging from 15% to 25%, depending on the stage of disease and assessment methods [45]. Insomnia has been consistently identified as both a core symptom and an independent predictor of depressive disorders in this population.

Recent longitudinal studies suggest a bidirectional relationship between insomnia and depression, whereby sleep disturbance increases vulnerability to depressive symptoms, while depression further exacerbates sleep disruption [46] and compromises quality of life and sleep patterns in patients with breast cancer [47,48]. This relationship is likely mediated by overlapping neurobiological pathways, including dysregulation of the HPA axis, alterations in monoaminergic systems, and inflammatory activation.

Several longitudinal studies in general oncology and survivorship populations suggest that insomnia symptoms may precede or predict subsequent depressive symptoms, supporting the hypothesis that sleep disturbance can contribute to later mood vulnerability. In breast cancer cohorts, however, the temporal evidence is more limited and often complicated by concurrent treatment effects, fatigue, pain, endocrine symptoms, and psychological distress. Therefore, although insomnia may represent an early and clinically modifiable marker of depressive risk, the claim that insomnia consistently precedes depression in breast cancer should be interpreted cautiously and requires further confirmation in prospective breast cancer-specific studies [10,46,47,48,49].

### 5.2. Anxiety, Hyperarousal, and Fear of Recurrence

Anxiety disorders and subclinical anxiety symptoms are highly prevalent among breast cancer patients, particularly in the early phases following diagnosis and during survivorship. Interestingly, women with breast cancer experience higher rates of anxiety disorders, while depression and sleep disorders show no gender disparity [50]. 

A central component of cancer-related anxiety is the fear of recurrence, a persistent and often intrusive concern that has been strongly associated with sleep disturbance [51]. Fear of recurrence has been indicated as a significant contributor to psychological distress and reduced quality of life [52], while anxiety is a predictor of quality of life in breast cancer survivors [53].

From a mechanistic perspective, anxiety contributes to insomnia through cognitive and physiological hyperarousal, characterized by rumination, heightened vigilance, and autonomic activation. This hyperarousal state interferes with sleep initiation and maintenance and may perpetuate chronic insomnia. Fear of recurrence seems not only associated with insomnia severity but may also mediate the relationship between psychological distress and sleep disruption, highlighting its central role in the psycho-oncological profile of breast cancer patients.

### 5.3. Fatigue and Energy Dysregulation

Cancer-related fatigue is one of the most distressing and persistent symptoms reported by breast cancer patients and is closely intertwined with insomnia. Although fatigue and sleep disturbance are distinct constructs, they frequently co-occur and may reinforce each other through shared biological mechanisms, particularly inflammation and circadian dysregulation [54].

It has been demonstrated that insomnia is a significant predictor of fatigue severity in breast cancer survivors, independent of disease status and treatment variables. Conversely, persistent fatigue may disrupt sleep–wake patterns, contributing to irregular sleep schedules and reduced sleep efficiency. Sleep traits (duration, insomnia, chronotype, snoring) may influence cancer risk and circadian disruption may have a role in carcinogenesis [35].

This bidirectional interaction supports the conceptualization of fatigue and insomnia as components of a unified energy regulation disturbance, rather than as separate clinical entities.

### 5.4. Pain, Cognitive Dysfunction, and Multisymptom Interaction

Pain is another key contributor to sleep disruption in breast cancer, particularly in patients undergoing active treatment or experiencing long-term treatment-related sequelae. Nocturnal pain can lead to frequent awakenings and fragmented sleep, while poor sleep may lower pain thresholds, creating a reciprocal amplification loop [55].

In addition, cognitive impairment, often referred to as “cancer-related cognitive dysfunction” or “chemo brain”, has been increasingly linked to sleep disturbances. Insomnia may impair attention, memory, and executive functioning through mechanisms involving disrupted synaptic plasticity and altered brain network activity [56,57].

The interaction between insomnia, pain, fatigue, and cognitive dysfunction underscores the complexity of symptom clustering and highlights the need for integrated assessment and management strategies.

### 5.5. A Transdiagnostic and Network-Based Perspective

The growing recognition of symptom clustering in breast cancer has led to the adoption of transdiagnostic models that conceptualize insomnia as a central node within a dynamic symptom network. In this framework, insomnia is not merely a secondary symptom but may actively contribute to the onset and maintenance of other symptoms, including depression, anxiety, and fatigue.

Evidence from symptom-cluster and network-based studies suggests that insomnia may function as a central or highly connected symptom within cancer-related symptom networks. In this framework, insomnia can contribute to the persistence or amplification of fatigue, emotional distress, pain sensitivity, and cognitive complaints, while these symptoms may in turn worsen sleep continuity. Nevertheless, the strength and directionality of these associations differ across studies, and not all available data are breast cancer-specific. Therefore, insomnia should be described as a potentially central and modifiable node rather than as a definitively established causal driver in all patients. This interpretation has clinical relevance because targeting insomnia may produce benefits extending beyond sleep, but it also highlights the need for longitudinal and mechanistic studies in breast cancer cohorts [43,44,58].

Network analysis studies in oncology populations suggest that targeting central symptoms, such as insomnia, may produce downstream improvements across multiple domains, offering a potentially more efficient therapeutic strategy [43,58].

This perspective is in line with emerging trends in precision medicine and psycho-oncology, emphasizing the importance of individualized, mechanism-based interventions that address shared underlying pathways rather than isolated symptoms.

Overall, the strong and consistent associations between insomnia and psychiatric comorbidities in breast cancer support its conceptualization as a core component of a broader neuropsychiatric symptom network. Recognizing and targeting insomnia within this framework may have significant implications for improving both psychological well-being and overall clinical outcomes. In the proposed transdiagnostic and network-based conceptualization of insomnia in breast cancer, sleep disturbance represents a central and potentially modifiable node within a broader neuropsychiatric symptom network.

## 6. Assessment and Diagnostic Considerations

Accurate assessment of insomnia in patients with breast cancer is essential for both clinical care and research comparability, as prevalence estimates vary substantially according to the instruments and diagnostic thresholds adopted [59,60]. In routine oncology settings, sleep disturbances are often identified through brief clinical questioning; however, structured assessment is preferable because it allows clinicians to distinguish generic sleep complaints from clinically significant insomnia symptoms or formal insomnia disorder. In this context, a multidimensional approach combining screening, patient-reported outcome measures, and diagnostic criteria is particularly relevant.

Among the most widely used self-report instruments, the Pittsburgh Sleep Quality Index (PSQI) is commonly employed to assess global sleep quality over the previous month [59]. The PSQI is especially useful in breast cancer populations because it captures multiple sleep domains, including sleep latency, duration, efficiency, disturbances, use of sleep medication, and daytime dysfunction. By contrast, the Insomnia Severity Index (ISI) is more specifically focused on insomnia symptom severity and related distress, providing a practical measure of perceived difficulties with sleep initiation, sleep maintenance, early morning awakenings, and associated daytime impairment [60]. While the PSQI may be more informative for a broad evaluation of sleep quality, the ISI may be particularly valuable for identifying clinically meaningful insomnia and for monitoring treatment response, especially in CBT-I studies.

Symptom scales should not be considered interchangeable with formal diagnostic criteria. Although self-report questionnaires are highly informative and feasible in oncological care, the diagnosis of insomnia disorder should remain grounded in established clinical criteria, such as those outlined in the DSM-5, which require persistent difficulty initiating or maintaining sleep, or early morning awakening, associated with significant distress or impairment despite adequate opportunity for sleep [61]. In breast cancer patients, this distinction is clinically important because sleep disruption may also reflect pain, vasomotor symptoms, treatment adverse effects, anxiety, depression, or circadian dysregulation, rather than insomnia disorder per se.

A clinically useful assessment strategy should therefore include not only standardized sleep instruments but also evaluation of comorbid psychiatric symptoms, cancer-related fatigue, pain, menopausal symptoms, medication use, and behavioral factors that may perpetuate sleep disturbance [24,25]. Sleep diaries, when feasible, may provide additional ecological information regarding sleep timing, variability, and nocturnal awakenings, while actigraphy can be considered in selected cases or research settings to better characterize circadian disruption and rest–activity patterns [62]. Overall, integrating validated tools such as the PSQI and ISI with DSM-5-based clinical evaluation may improve diagnostic precision, facilitate early identification of high-risk patients, and support more personalized treatment planning in breast cancer care.

## 7. Treatment Approaches and Clinical Management

The management of insomnia in breast cancer requires a multimodal and interdisciplinary approach, integrating behavioral, pharmacological, and emerging interventions. Given the multifactorial pathophysiology of insomnia in this population, treatment strategies should be tailored to address both the underlying biological mechanisms and the associated psychological and behavioral components.

### 7.1. Cognitive Behavioral Therapy for Insomnia (CBT-I)

Cognitive behavioral therapy for insomnia (CBT-I) is currently considered the first-line treatment for chronic insomnia, including in oncological populations. CBT-I is a structured, evidence-based intervention targeting maladaptive sleep behaviors and dysfunctional cognitive processes through techniques such as stimulus control, sleep restriction, cognitive restructuring, and relaxation training.

Recent randomized controlled trials and meta-analyses have demonstrated that CBT-I is highly effective in breast cancer patients, leading to significant improvements in sleep quality, insomnia severity, and related symptoms such as fatigue and depression [63,64]. These benefits appear to be sustained over time, supporting the durability of CBT-I effects in survivorship populations [65,66].

Importantly, randomized trials and meta-analyses in breast cancer and broader cancer survivor populations indicate that CBT-I improves insomnia severity and sleep quality and may also reduce fatigue and depressive symptoms. These findings support the clinical value of early insomnia treatment as part of integrated psycho-oncological care. Current evidence more strongly supports symptom improvement than formal prevention of incident major depressive disorder. Thus, early treatment of insomnia should be framed as a plausible strategy to reduce depressive vulnerability and improve psychological outcomes, rather than as an established depression-prevention intervention in breast cancer patients [3,63,64,65,66,67].

Digital and internet-delivered CBT-I interventions have gained increasing attention in recent years, offering scalable and accessible treatment options. Studies suggest that these approaches are comparable in efficacy to face-to-face therapy and may be particularly valuable for patients with limited access to specialized care [67].

Despite strong evidence supporting CBT-I, its implementation in routine oncology care remains limited, highlighting the need for greater integration of behavioral sleep medicine into cancer survivorship programs.

### 7.2. Pharmacological Treatments

Pharmacological interventions are frequently used in the management of insomnia in patients with cancer, although they are generally recommended as short-term or adjunctive treatments rather than first-line options.

Commonly prescribed agents include benzodiazepines, non-benzodiazepine hypnotics (e.g., zolpidem), melatonin receptor agonists, and certain antidepressants with sedative properties. While these medications may provide rapid symptomatic relief, their use is associated with potential risks, including tolerance, dependence, cognitive impairment, and interactions with oncological treatments [40].

Melatonin has attracted particular interest due to its role in circadian regulation and potential oncostatic properties. It has also been considered an option for patients with breast cancer experiencing cancer-related fatigue [68]. Recent studies suggest that melatonin supplementation may improve sleep quality and circadian alignment in cancer patients, although evidence remains heterogeneous and further research is needed to define optimal dosing and patient selection [69,70]. Pharmacological treatment should be individualized, carefully monitored, and ideally combined with behavioral interventions to maximize efficacy and minimize adverse effects.

### 7.3. Integrative and Non-Pharmacological Interventions

A growing body of literature supports the role of integrative approaches in the management of insomnia in breast cancer. These include mindfulness-based interventions, yoga, physical activity, acupuncture, and relaxation techniques.

Mindfulness-based stress reduction (MBSR) and related interventions have been shown to improve sleep quality, reduce psychological distress, and modulate stress-related physiological pathways, including cortisol and inflammatory markers [71,72,73]. Similarly, structured physical activity programs have demonstrated beneficial effects on both sleep and fatigue, likely mediated by improvements in circadian regulation and mood [74].

Acupuncture and other complementary therapies have also been explored, with some studies suggesting modest improvements in insomnia symptoms, although evidence remains variable and methodological heterogeneity limits definitive conclusions [75,76].

These interventions may be particularly valuable as adjunctive treatments, especially in patients who prefer non-pharmacological approaches or have contraindications to medication.

### 7.4. Toward Personalized and Mechanism-Based Interventions

Emerging research emphasizes the importance of personalized approaches to insomnia treatment in breast cancer, taking into account individual variability in symptom profiles, biological mechanisms, and psychological factors [77,78].

From a neuropsychiatric perspective, targeting shared underlying pathways (inflammation, circadian disruption, and stress-related neural circuits) may offer a more effective strategy than treating symptoms in isolation. For example, interventions aimed at reducing inflammation or restoring circadian alignment may simultaneously improve sleep, fatigue, and mood. In addition, the identification of specific insomnia phenotypes (e.g., hyperarousal-driven vs. circadian-disruption-driven insomnia) may facilitate more precise and targeted interventions, in line with the principles of precision medicine.

In summary, the treatment of insomnia in breast cancer should prioritize evidence-based behavioral interventions, particularly CBT-I, while incorporating pharmacological and integrative approaches when appropriate. A shift toward personalized, mechanism-informed care has the potential to improve outcomes not only for sleep but also for the broader neuropsychiatric symptom burden associated with breast cancer.

A comprehensive overview of the main domains involved in insomnia in breast cancer is presented in Table 1.

## 8. Discussion

This narrative review highlights insomnia as a highly prevalent and clinically significant condition in breast cancer, extending far beyond a secondary or transient symptom. The evidence synthesized across epidemiological, neurobiological, and clinical domains supports a reconceptualization of insomnia as a core component of a broader neuropsychiatric and stress-related symptom network.

One of the central findings emerging from this review is the consistent co-occurrence of insomnia with depression, anxiety, fatigue, pain, and cognitive dysfunction. This clustering suggests that insomnia may not only reflect shared underlying mechanisms but also actively contribute to symptom amplification and persistence [79]. From a clinical perspective, this has important implications, as it challenges the traditional symptom-by-symptom management approach and supports the adoption of more integrated, transdiagnostic models of care [80,81].

The neurobiological evidence further reinforces this perspective. Dysregulation of the HPA axis, chronic low-grade inflammation, and circadian rhythm disruption appear to converge in shaping both sleep disturbances and broader psychological vulnerability. These overlapping pathways provide a plausible mechanistic framework linking insomnia to mood disorders, fatigue, and potentially even oncological outcomes. Importantly, the bidirectional relationship between sleep and these biological systems suggests the presence of self-reinforcing loops, in which insomnia both results from and contributes to physiological and psychological dysregulation.

Another key aspect concerns the persistence of insomnia across the cancer trajectory. Unlike acute treatment-related side effects, sleep disturbances often remain stable or even worsen during survivorship, indicating that insomnia should be conceptualized as a chronic condition requiring long-term management strategies. This has significant implications for survivorship care models, which frequently underemphasize sleep and mental health despite their central role in quality of life and functional recovery.

Despite robust evidence supporting the efficacy of cognitive behavioral therapy for insomnia (CBT-I), its implementation in routine oncology practice remains limited. Barriers include lack of trained professionals, limited integration between oncology and behavioral health services, and insufficient screening for sleep disorders. The growing availability of digital CBT-I platforms offers a promising avenue to bridge this gap, although further research is needed to optimize adherence and personalization.

This review also underscores the need for a more personalized and mechanism-based approach to insomnia in breast cancer. The heterogeneity of clinical presentations suggests that different patients may exhibit distinct pathophysiological profiles, such as inflammation-driven, circadian-driven, or hyperarousal-driven insomnia. Identifying these phenotypes could allow for more targeted and effective interventions, in line with the broader paradigm of precision medicine.

Several limitations should be acknowledged. As a narrative review, this work does not provide a quantitative synthesis of the evidence and may be subject to selection bias. Although priority was given to recent and methodologically robust studies, heterogeneity in study designs, assessment tools, and patient populations limits direct comparability across findings. Future research would benefit from standardized definitions of insomnia, longitudinal designs, and integration of biological and psychological measures.

This review suggests a shift from a symptom-centered to a systems-based understanding of insomnia in breast cancer, emphasizing its role as a central and potentially modifiable factor within a complex neuropsychiatric landscape.

## 9. Conclusions

Insomnia is a highly prevalent, persistent, and clinically consequential condition in patients with breast cancer, yet it remains insufficiently recognized and managed in routine care. Accumulating evidence indicates that insomnia should not be viewed as an isolated sleep disorder, but rather as a neuropsychiatric condition embedded within a broader network of stress-related symptoms and biological dysregulation.

The integration of neuroendocrine, inflammatory, circadian, and psychological mechanisms provides a comprehensive framework for understanding the multifactorial nature of insomnia in this population. Within this framework, insomnia emerges as both a marker and a driver of vulnerability, with potential implications for quality of life, functional outcomes, and possibly disease trajectory.

From a clinical standpoint, systematic screening for sleep disturbances should become a standard component of breast cancer care, particularly during survivorship. Cognitive behavioral therapy for insomnia should be considered the first-line treatment, with pharmacological and integrative approaches used in a complementary and individualized manner.

Future research should focus on the identification of mechanistic subtypes of insomnia, the development of personalized treatment strategies, and the integration of sleep-focused interventions into multidisciplinary oncology care pathways. For example, there is promising evidence that specific butyrate-producing gut bacteria, rather than overall microbial diversity, showed temporal associations with sleep quality in breast cancer patients undergoing chemotherapy and these findings suggest that targeted microbiota interventions warrant further investigation as potential strategies for improving sleep quality during cancer treatment [82].

**Figure 5:**
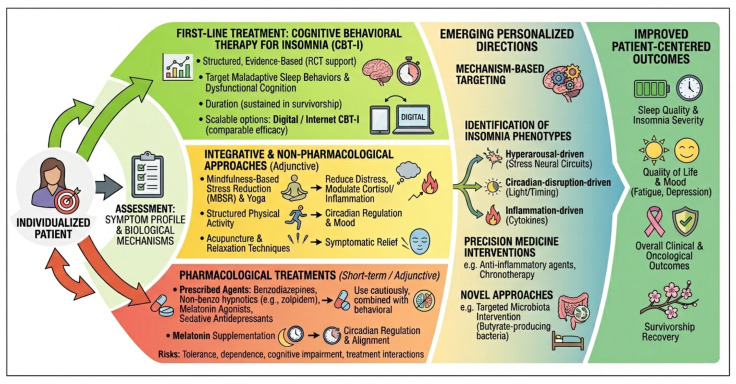
Integrated conceptual model of insomnia in breast cancer. Note: This integrative diagram outlines a patient-centered approach to managing sleep disturbances. The (**left**) panel emphasizes the transition from an Individualized Patient assessment of symptom profiles and biological mechanisms to tailored interventions. The (**central**) panel details the three main therapeutic pillars: First-line Treatment (CBT-I), focusing on evidence-based behavioral and cognitive restructuring, including digital/scalable options; Integrative & Non-Pharmacological Approaches, such as MBSR, yoga, and physical activity to modulate stress and inflammation; Pharmacological Treatments, highlighting the cautious use of hypnotics and melatonin for circadian alignment. The (**right**) panel represents Emerging Personalized Directions, where identifying specific insomnia phenotypes (e.g., hyperarousal-driven, circadian-disruption-driven, or inflammation-driven) allows for precision medicine and novel approaches like targeted microbiota interventions. The model culminates in Improved Patient-Centered Outcomes, encompassing sleep quality, mood, quality of life, and overall oncological recovery during survivorship. Abbreviations: CBT-I: Cognitive Behavioral Therapy for Insomnia; MBSR: Mindfulness-Based Stress Reduction (Mindfulness Disposition); RCT: Randomized Controlled Trial (Structural Activity).

Insomnia in breast cancer should not be considered as a secondary symptom but a central neuropsychiatric condition within a complex symptom network; recognizing and targeting it may substantially improve patient outcomes across multiple domains. To consolidate the findings of this review and provide a comprehensive visual summary, an integrated conceptual model is presented in Figure 5, illustrating the interplay between epidemiology, biological mechanisms, symptom networks, and multimodal treatment strategies for insomnia in breast cancer.

Addressing insomnia as a central therapeutic target may offer a unique opportunity to improve both psychological well-being and overall clinical outcomes in patients with breast cancer.

## Figures and Tables

**Table 1:** Overview of Insomnia in Breast Cancer: Epidemiology, Mechanisms, Clinical Correlates, and Treatment Approaches.

Domain	Key Findings	Clinical Implications
Prevalence and Epidemiology	Insomnia affects approximately 30–70% of breast cancer patients; poor sleep quality reported in up to 60% of survivors. Symptoms may persist years after treatment completion.	Insomnia should be considered a chronic condition in survivorship and routinely screened in clinical practice.
Neuroendocrine Mechanisms (HPA Axis)	Chronic stress leads to HPA axis dysregulation, with elevated cortisol levels and flattened diurnal rhythms.	Targeting stress regulation may improve both sleep and psychological outcomes.
Inflammatory Pathways	Increased levels of IL-6, TNF-α, and CRP are associated with insomnia, fatigue, and depression. Bidirectional relationship between sleep and inflammation.	Anti-inflammatory and behavioral interventions may have synergistic effects.
Circadian Disruption	Altered circadian rhythms due to cancer treatments, light exposure, and behavioral changes; impaired melatonin secretion.	Chronotherapy and light-based interventions may represent targeted treatment options.
Treatment-Related Factors	Chemotherapy, endocrine therapy, and menopausal symptoms (e.g., hot flashes) contribute to sleep disruption.	Long-term monitoring of sleep is needed, even after treatment completion.
Psychiatric Comorbidity	Strong associations with depression, anxiety, fear of recurrence, and cognitive dysfunction.	Integrated psycho-oncological assessment is essential.
Symptom Clusters	Insomnia co-occurs with fatigue, pain, and mood symptoms, forming a multidimensional symptom network.	Targeting insomnia may produce downstream benefits across multiple symptoms.
Behavioral Treatment (CBT-I)	First-line treatment with strong evidence for efficacy in cancer populations; improves sleep, fatigue, and mood.	Should be implemented as standard care; digital CBT-I may improve accessibility.
Pharmacological Treatment	Short-term benefits from hypnotics, antidepressants, and melatonin; risks include dependence and side effects.	Use cautiously and in combination with behavioral approaches.
Integrative Approaches	Mindfulness, physical activity, yoga, and acupuncture show beneficial effects on sleep and distress.	Useful as adjunctive therapies, especially for patient-centered care.
Future Directions	Emerging focus on personalized, mechanism-based treatment (e.g., inflammation-driven or circadian-driven insomnia).	Precision medicine approaches may optimize outcomes and resource allocation.

Abbreviations: CBT-I, Cognitive Behavioral Therapy for Insomnia; HPA axis, Hypothalamic–Pituitary–Adrenal axis; IL-6, Interleukin-6; TNF-α, Tumor Necrosis Factor-alpha; CRP, C-reactive protein.

## Data Availability

No new data were created.
